# Haemonchosis in Small Ruminants Under Traditional Husbandry Systems in Apac District, Northern Uganda

**DOI:** 10.1155/2024/8812142

**Published:** 2024-10-23

**Authors:** Charles Dickens Opeto, Harriet Angwech, Acaye Ongwech, Benard Abola, Steven Odongo, Geoffrey M. Malinga

**Affiliations:** ^1^Department of Biology, Faculty of Science, Gulu University, PO Box 166, Gulu, Uganda; ^2^Department of Veterinary, Directorate of Production and Marketing, Kwania District, PO Box 1, Uganda; ^3^Department of Chemistry, Faculty of Science, Gulu University, PO Box 166, Gulu, Uganda; ^4^Department of Mathematics, Faculty of Science, Gulu University, PO Box 166, Gulu, Uganda; ^5^Department of Biotechnical and Diagnostic Sciences, College of Veterinary Medicine, Animal Resources and Biosecurity (COVAB), Makerere University, PO Box 7062, Kampala, Uganda; ^6^Center for Biosecurity and Global Health, College of Veterinary Medicine, Animal Resources and Biosecurity (COVAB), Makerere University, PO Box 7062, Kampala, Uganda

**Keywords:** faecal egg count, *Haemonchus contortus*, intensity of infection, northern Uganda, prevalence, risk factors

## Abstract

*Haemonchus contortus* is one of the most common and fatal pathogenic gastrointestinal nematodes of small ruminants causing significant economic losses, particularly in low-income countries. A cross-sectional study was carried out in randomly selected small ruminants kept under traditional husbandry systems in the Apac district (now split into Apac and Kwania districts) in northern Uganda to assess the prevalence, intensity, and associated risk factors of *H. contortus* infection from May 2018 to January 2019. Faecal samples were collected from a total of 768 randomly selected small ruminants (384 sheep and 384 goats) and examined for parasites using the floatation method and coproculture. The overall prevalence of *H. contortus* in small ruminants was 73.3% (563/768) by faecal egg count and 70.6% (542/768) by coproculture. The mean number of eggs per gram of faeces in small ruminants was 2046 ± 107 and differed significantly across the host animals ranging from 1729 ± 120 (mean ± SE) in goats to 2364 ± 176 in sheep. Significant predictors of infection were the origin of study animals, animal species, breed of animal, age of study animal, faecal consistency, lactation, multispecies grazing, grazing method, and anthelmintic use. The findings of our study provide information on the current status of *H. contortus* infections in goats and sheep under traditional husbandry systems in Uganda which are critical in designing effective control strategies for the disease.

## 1. Introduction

Small ruminants (notably goats and sheep) are among Uganda's top economically valuable livestock [1] due to their hardiness and ability to produce many offspring over a relatively short duration [2]. There are about 18 million small ruminants in the country [3], playing important roles in the socioeconomic well-being of smallholder farmers through the provision of ready cash, meat, hides, milk, and payment of dowries [4]. They also provide intangible benefits such as savings and insurance against emergencies and crop failures [5, 6]. The contribution to Uganda's total GDP of livestock production is about 4.2% [7]. The recent reintegration and opening of free trade among the East African communities, including Southern Sudan, and increasing domestic demand imply a need to increase the scale of small ruminants' production [3]. However, gastrointestinal parasite infection remains a major constraint hampering their full productivity.

Haemonchosis, a disease caused by *Haemonchus contortus* (also called barber pole worm), is one of the leading economically important and highly pathogenic diseases of small ruminants ([8]: [9]), causing production losses in small ruminant production in Uganda [10]. The economic losses are primarily through increased morbidities and mortalities, particularly in severely infested animals, and the cost of buying drugs and treatment [11]. *H*. *contortus* is a blood-sucking nematode that primarily lives in the abomasum of small ruminants. The parasite has a worldwide distribution with high concentrations in the tropics and subtropics, where weather conditions are conducive [12]. A single worm can suck approximately 0.05 mL of blood daily through either ingestion or seepage from lesions [13]. The females can produce more than 5000 eggs per day which are shed in the host's faeces onto the pasture [14]. Infected animals may have hemorrhagic anemia due to the leaking of blood by adult worms and fourth-stage larvae from the abomasal wall [11], experience loss of appetite, oedema, and retarded growth [14].

Despite the economic importance of this parasite and the high population of small ruminants in the country, there is currently limited documented data regarding the epidemiology of small ruminant haemonchosis. There are invariable reports of prevalence data of *H. contortus* infection in small ruminants in the country, ranging from as low as 12.8% in the neighbouring district of Nakasongola [15], 36.6% in goats in Sironko district [16], and 56% in central Uganda [17]. In Ethiopia, Bitew, Amde, and Belachew [18] recorded a prevalence of 75.9% and 55.9% in sheep and goats, respectively. Host factors (e.g., species, age, sex, and breed), epidemiological factors such as body condition and origin (collection sites), and environmental and management factors (e.g., agroecological conditions, weather conditions, frequency of deworming, quantity and quality of pasture, and animal husbandry practices) may determine the onset of infections, prevalence, incidence, and severity of the disease in affected animals [16, 19–21]. There is currently very limited information on the burden of haemonchosis in sheep and goats kept under smallholder traditional animal husbandry in northern Uganda. Data on the specific risk factors are also scarce. To develop effective control and prevention strategies for *H. contortus* infection of small ruminants in the smallholder systems, it is important to identify specific risk factors unique to this area. Therefore, the objectives of this study were to determine the prevalence, intensity, and risk factors of haemonchosis in small ruminants (goats and sheep) kept under the traditional husbandry practices in Apac district, northern Uganda.

## 2. Materials and Methods

### 2.1. Description of Study Area

**Figure 1:**
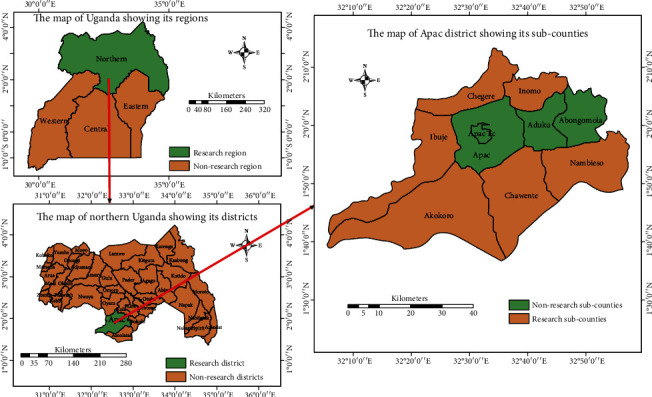
Location of the subcounties in Apac district, Uganda, that were sampled.

The study was conducted in Apac district (now split into Kwania and Apac districts) located 320 km from Kampala, the capital city of Uganda, from May 2018 to January 2019. Of the 10 subcounties in the district, six were included in the study (Figure 1). Geographically, the district lies between longitudes 32° E and 34° E and latitudes 2° N and 3° N and has an average altitude of 1150 mm above sea level and is bordered by L. Kwania to the south and southeast. The area is predominantly dry savannah, with a bimodal rainfall pattern, characterized by a short rainy season (April to November), rainfall peaks in April and August, and a dry season from December to March [3]. The study area receives a mean rainfall of 1330 mm annually. The average monthly minimum and maximum temperatures were 17°C in April and August and 29°C in December to March, respectively [3]. These conditions favour the proliferation of many nematode parasites including *H. contortus*. The district covers a total area of 2847 km^2^, of which 9% is open swamps and water, while 15% is forest, with 2970 km^2^ for human settlement and 2524 km^2^ suitable for arable farming [22]. The livestock population of Apac district is estimated to be 279,649 goats and 45,967 sheep, mostly local breeds [3], that are known to be well adapted to harsh environmental conditions and limited feed and are also more resistant to diseases. The predominant goat species are the East African small goats and their crosses with the exotic Boer goats. While for sheep, the Dorset horn breed cross and the Ankole fat–tailed sheep were the breeds encountered. Small ruminants are reared on natural grass pastures and shrubs under the traditional grazing system where animals graze freely in open communal grazing grounds alongside other animals such as cattle and donkeys or are tethered with very minimal access to veterinary services. The animals are primarily reared in small flocks of four to eight or less per household and are used mainly for income generation and meat production.

### 2.2. Study Animals

The study animals were sheep and goats of different sexes, ages, breeds, and body condition scores (BCSs) kept on natural pasture and under the traditional husbandry management systems with no or minimal access to veterinary services.

### 2.3. Study Design

A cross-sectional study was carried out to determine the prevalence, intensity, and associated risk factors of *H. contortus* infection in sheep and goats owned by resource-poor farmers in Apac district. Before examining each animal for the presence or absence of parasites, data about the age, BCSs, and animal origin was collected from the livestock owners using a semistructured questionnaire and by animal inspection.

### 2.4. Sample Size Determination

The sample size (*n*) for the study animals was determined according to Krejcie and Morgan's [23] formula, considering a 95% confidence interval (CI), 5% desired absolute precision, and 50% expected level of prevalence of *H. contortus* infection among sheep and goats in the study area since there was no previous study specifically in the study area. Accordingly, a total of 768 small ruminants comprising 384 sheep and 384 goats from 220 households were randomly selected and examined for *H. contortus* infection.

### 2.5. Selection of Study Area and Sample Collection

A multistage sampling procedure was used to recruit small ruminants for the study. First, Apac district was divided into two agroecological zones, that is, the North Eastern Savannah Grassland (NESG) in the northern part and the Kyoga plains (KPs) in the southern part [24]. From each agroecological zone, three subcounties—Chawente, Nambieso, and Akokoro in the KP zone and Inomo, Chegere, and Ibuje subcounties in the NESG zone—were randomly selected. In each of the selected subcounties, two parishes were randomly selected, totaling 12 parishes. Finally, small ruminants (*n* = 768) comprising sheep (*n* = 384) and goats (*n* = 384) were randomly and proportionally selected from the parishes for the study.

For the faecal sample collection, 10–15 g of faeces per animal was scooped from the rectum using a sterile gloved index finger as described by Soulsby [25]. The collected sample was individually placed into a labelled plastic bottle and tightly closed with a screw cap. The samples were transported in an ice cool box to the livestock laboratory of Kachwekano Zonal Agricultural Research Development Institute (ZARDI), Uganda, on the same day of collection and then kept at +4°C until processing for coproculture and microscopy. Faecal samples kept for more than 2 days prior to copromicroscopic analysis were preserved in 10% formalin to prevent the eggs from developing and hatching. During sampling, data concerning sex, age, breed, faecal consistency/water content of faeces, origin (subcounty), housing, presence of other animals or multispecies grazing, anthelmintic treatment or deworming (yes/no), and management practices in general were recorded for each sampled animal from farm owners. The age of the animals was determined based on the farmers' response and dentition inspection as young (≤ 1 year) and adult (> 1 year). To determine the BCS of each animal, a visual assessment of the carcass was performed based on the level of fat deposition and muscles, and BCS was grouped according to Morgan et al. [26] as 1 (*poor*), 2 (*average* or *medium*), and 3 (*good*).

### 2.6. Parasitological Examination of Faecal Samples

At the laboratory, all the faecal samples were crushed thoroughly, and 4 g of each crushed faecal sample from each animal was mixed with 60 mL of NaCl solution (floatation fluid) and blended. After obtaining a homogenous mixture, it was sieved into a beaker. A few drops of amyl alcohol (3–6) were added to treat bubbles in the mixture. The disposable pipettes were used to draw a few milliliters to fill the two chambers of the McMaster slides, which were viewed under the microscope at ×10 objective as recommended by Dryden et al. [27]. Identification of parasite eggs was based on morphological characteristics. Floating parasite eggs were then counted using a laboratory cell counter, and each number was multiplied by a factor of 50 to give an approximate number of eggs per gram (EPG) of fresh faeces [28]. The intensity of infection was considered light (50–800 EPG), moderate (800–1200 EPG), and heavy (> 1200 EPG) as described by Hansen and Perry [29].

### 2.7. Faecal Culture and Larval Identification

To prepare faecal culture, approximately 5–10 g of every faecal sample was stirred using a spoon to make it crumbly. In case it was too dry, water was added to make it moist, and if it was too wet, charcoal was added until the correct consistency was obtained. Thereafter, the sample was transferred to a sterile plastic container and incubated at room temperature for 14–21 days. Water was added regularly after every 1–2 days to the cultures. After incubation at room temperature, the larva (third stage) was isolated from the faecal sample using the Baermann technique [30]. The isolated larva was examined under ×10 magnification. Morphological larval identification followed the keys of Hansen and Perry [29].

### 2.8. Data Analyses

All data collected were coded and entered in Microsoft Excel Spread Sheet and subsequently used to analyse simple descriptive statistics like percentages, proportions, and frequency distributions of the data. The prevalence of *H. contortus* was calculated for all data by dividing the number of infected animals by the total number of animals examined. The 15 factors thought to be associated with the prevalence of *H. contortus* infection (animal species, breed, sex, age, BCS, faecal consistency, coinfections, physiological status, and animal origin, as well as management-related factors) were analyzed first with chi-square and binary logistic regression. Then, nine variables with *p* values less than 0.05 in the analysis were further subjected to the final stepwise multivariable logistic regression analysis to determine risk factors associated with *H. contortus*. It should be noted that multicollinearity was examined for significant variables, or those with a *p* value of 0.05 or less. Only variables that passed the test as a result were added for the multivariable analysis. The crude odds ratio (cOR) and adjusted odds ratio (aOR) of the prevalence of *H. contortus* with 95% CI and a significance threshold of 5% were used to describe the associations. To examine whether the intensity of infection differed among the host and management parameters studied, a nonparametric Kruskal–Wallis test was used, followed by Dunn's test for post hoc pairwise multiple comparisons. All analyses were done using IBM SPSS for Windows Version 25 (IBM, Armonk, NY, United States).

## 3. Results

### 3.1. General Characteristics of Study Households

Of the small ruminant farmers examined, 59.2% were males with little or no formal education, 48.2% at the primary level, while only 29.9% attained secondary level (Table 1). The mean age of respondents was 45.6 years, with the majority (32.2%) within the age group of 30–40 years. Only about 34% of the farmers dewormed their animals. The most commonly used dewormers were mainly from the benzimidazole class such as fenbendazole, albendazole, and oxibendazole. A few farmers used levamisole (an imidazothiazole) and a combination of ivermectin and albendazole (wormicid) too.

### 3.2. Prevalence of *H. contortus* and Associated Risk Factors in Small Ruminants

Overall, 73.3% (95% CI: 70.0–76.4) of the small ruminants studied in Apac district (*n* = 768) had eggs and 70.6% (95% CI: 67.2–73.8) had larvae of *H. contortus* in their faeces. Besides *H. contortus*, other nematode larvae recovered were *Oesophagostomum* (17.5%), *Strongyloides* (5%), *Trichostrongylus* spp. (5%), and *Cooperia* spp. (2.5%). While 57% of *H. contortus* infections occurred as mixed infections with *Eimeria* spp. based on copromicroscopy. From chi-square analyses, the following factors were not significantly associated with haemonchosis and therefore dropped from the subsequent analyses: sex of study animal, BCS, pregnancy status, coinfection, size of grazing land, pasture quality, farmers' level of education, and agroecological zone (Table 1). Multiple logistic regression revealed that the only factors significantly associated with the prevalence of *H. contortus* in small ruminants in the district were the origin of study animals (subcounty), animal species, breed of animal, age of study animal, faecal consistency, lactation, multispecies grazing, grazing system, and anthelmintic use (Table 2). We found a significant association between *H. contortus* and origin of study animals (*χ*^2^ = 12.9, *p* = 0.025). Small ruminants from Akokoro, Nambieso, and Chegere subcounties were almost twice ([aOR] 1.87, 95% [CI]: 1.08–3.23) and thrice more at risk of *H. contortus* infection ([aOR] 2.5, 95% [CI]: 1.4–4.45) than those from Ibuje subcounty. There was a significant association between *H. contortus* infection and animal species (*χ*^2^ = 12.3, *p* = 0.001). Goats had a 36% lower chance of *H. contortus* infection than sheep ([aOR]: 0.64; 95% [CI]: 0.43–0.95). Similarly, a significant association (*χ*^2^ = 3.99, *p* = 0.046) existed between small ruminant breeds and the prevalence of *H. contortus* infections. Local breeds were 53% less likely to be infected with *H. contortus* than the crossbreeds ([aOR] 0.47; 95% [CI]: 0.26–0.88). In addition, we found a significant association between the age of the animals and *H. contortus* (*χ*^2^ = 5.07, *p* = 0.02). Younger small ruminants had approximately twice higher odds of infection ([aOR] 1.64; 95% [CI]: 1.11–2.44) compared to adult animals. Surprisingly, a significant association (*χ*^2^ = 21.6, *p* ≤ 0.001) existed between faecal consistency and prevalence of *H. contortus* infection. Small ruminants with normal faeces were 48% less likely to be shading *H. contortus* eggs than those passing soft stool ([aOR] 0.52; 95% [CI]: 0.36–0.74). Furthermore, in farms where goats, sheep, and cattle were not grazed together, there was a 59% lower risk of *H. contortus* infection than grazing mixed species together ([aOR] 0.41; 95% [CI]: 0.29–0.58). Finally, there were significant associations between the infection and deworming (*χ*^2^ = 20.76, *p* ≤ 0.001) as well as the grazing system (*χ*^2^ = 10.6, *p* ≤ 0.001). The risk of haemonchosis was twofold higher in small ruminants not dewormed as compared to those that were dewormed ([aOR] 1.97; 95% [CI]: 1.37–2.83) while tethering significantly increased the odds of infection by almost twofold ([aOR] 1.68; 95% [CI]: 1.17–2.42).

### 3.3. Intensity of *H. contortus* Infection in Small Ruminants

**Figure 2:**
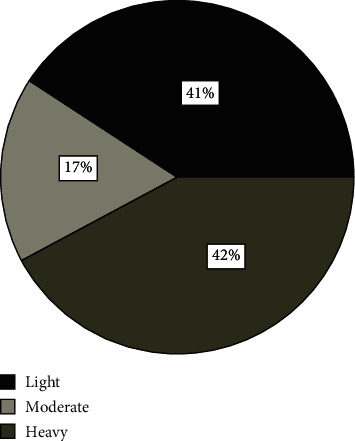
Intensity of *H. contortus* in the study area (percentage of animals in the different categories).

**Figure 3:**
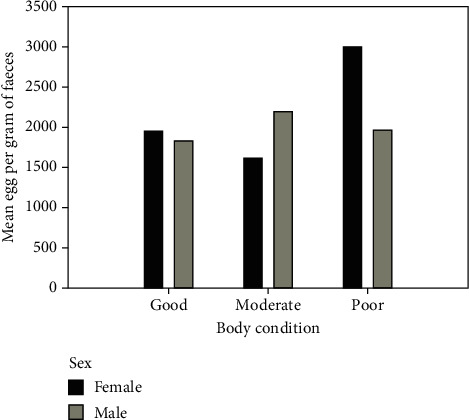
Intensity (EPG) of *Haemonchus contortus* by body condition and sex of the study animals.

**Figure 4:**
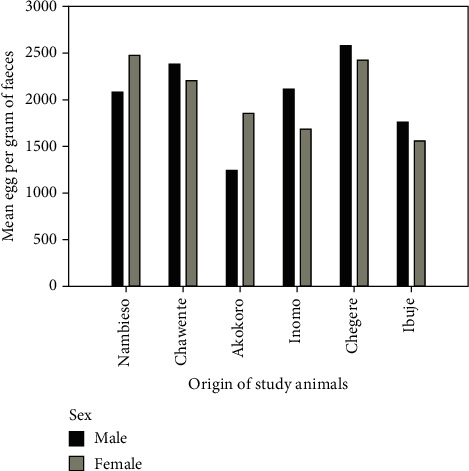
Intensity (EPG) of *Haemonchus contortus* by place of origin (subcounties) and sex of animals.

The intensity of infection measured in EPG of faeces was used to estimate worm burden since the egg counts tend to be correlated with worm burden [31, 32]. Of the 768 small ruminants examined, 42.1% (*n* = 237) were heavily infested, with *H. contortus*, 17.2% (*n* = 97) were moderately infested, and 40.7% (*n* = 229) were lightly infested (Figure 2). Intensity (EPG of faeces) of *H. contortus* differed significantly across the hosts' species, husbandry practices such as multispecies grazing, grazing system, and anthelmintic use, as well as the physiological status of animals. There was a significant difference in the intensity of infection by body condition (Kruskal–Wallis test, *H* = 12.8; df 2; *p* = 0.002). Poorly conditioned animals were more heavily infested than the good or moderately conditioned small ruminants (*p* ≤ 0.001) while no significant difference was observed between good and moderately conditioned animals (*p* > 0.05). However, when sex was considered, females had 41% more EPG compared to the male animals among the poor-conditioned animals (Figure 3). Worm burden was also significantly higher in sheep than in goats (*H* = 5.4; df 1; *p* = 0.02). As expected, worm intensity was higher in animals that were not dewormed (*H* = 9.8; df 1; *p* = 0.002). Where farmers were trained and therefore able to treat their animals by themselves, the worm burden was also low (*H* = 10.2; df 2; *p* = 0.006) compared to soliciting help from veterinarians and friends. The egg count per gram of faeces was higher in animals passing soft stool than in those with normal faecal consistency (*H* = 9.8; df 1; *p* = 0.002). Similarly, the grazing system significantly affected the intensity of *H. contortus* in small ruminants, with tethered animals presenting with higher worm burden than those reared on the free range (*H* = 3.8; df 1; *p* = 0.05). The intensity of infection was also affected by lactation in that lactating animals had a higher worm burden (*H* = 6.4; df 1; *p* = 0.011). Conversely, no significant differences were observed by sex, pregnancy status, breed, and origin of sampled animals (*p* > 0.05); however, when origin was contrasted with sex, there was a slight difference between males and females only for Akokoro (38% [597.86]) and Nambieso (16.8% [384.11]; Figure 4).

## 4. Discussion

### 4.1. Prevalence of *H. contortus* in Small Ruminants

In this study, a prevalence of 73.3% and 70.6% were observed using faecal egg identification and coproculture techniques, respectively. As reported in another study, this high prevalence can partly explain the low productivity of small ruminants in Apac [17]. This high nematode prevalence ranging from 53.7% to 76% has been reported before in Uganda [17, 33] and in Africa in general [5, 20, 34, 35] in small ruminants. In Uganda, small ruminants are mainly raised under the traditional management system, mostly in low-lying pastures where animals are either tethered or freely grazed with limited veterinary care. The high prevalence of *H. contortus* found in this study might be due to suitable climatic conditions (e.g., sufficient rainfall, sunlight, temperatures, humidity and soil moisture during the study period coupled with high stocking density, inadequate nutritional status, year-round communal grazing, and lack of awareness by the community) [21]. These climatic conditions favour the development and survival of viable eggs and infective stages within faecal pellets in herbage since *H. contortus* has a high biopotential and can establish very rapidly as long as the prevailing conditions are favourable. The prevalence of *H. contortus* in small ruminants differed by origin. Animals in Chegere, Akokoro, and Nambieso subcounties had a significantly higher prevalence of *H. contortus* infection than those from other subcounties. These findings can be explained by the variations in climatic conditions between the different subcounties. Akokoro and Nambieso subcounties have very extensive hydrological systems; hence, flooding and swamp formation are likely to cause high infection rates. Besides, the Chegere subcounty is adjacent to an open swamp, providing humidity and soil moisture required for the development of viable eggs of *H. contortus* within faecal pellet herbage.

A significantly higher proportion of sheep than goats had *H. contortus* eggs in their faeces indicating that sheep are more susceptible to *H. contortus* infections than goats. This observation is consistent with the findings of earlier work in Ethiopia [19]. The higher prevalence in sheep than in goats arises because sheep tend to graze very close to the ground where infective nematode larvae are prevalent, increasing their chances of picking infective larval stages, while goats are predominantly browsers, feeding on shrubs and small trees above the ground, where infective larvae are comparatively fewer [19].

### 4.2. Intensity of *H. contortus* in Small Ruminants

The intensity of parasitic infection depends mainly upon the age of the host, breed, parasitic species involved, and epidemiological patterns, including husbandry practices and animal physiological status [36]. In this study, the overall intensity of *H. contortus* showed that the EPG counts differed across the hosts, breed, husbandry practices, and physiological status of animals. However, the overall mean number of EPG of faeces in small ruminants was 2046 ± 107, indicating a high level of *H. contortus* infection in the district. The relatively high intensity of haemonchosis observed could also be associated with suitable environmental conditions during the study period which favoured the development of eggs and free-living stages of the parasites. In general, the overall intensity of *H. contortus* infection in this study was high and consistent with a previous report by Abdo [37]. This high intensity of *H. contortus* could be regarded as a problem affecting the productivity of small ruminants, especially in the traditional farming systems (mixed livestock crop) similar to that practiced in Apac district, where farmers do not provide nutritional supplements or invest in buying drugs for controlling the helminths [38].

Although factors such as the body condition of small ruminants were not significant correlates of *H. contortus* prevalence in the study, we found a significant relationship with worm burden (i.e., faecal egg count). Poorly conditioned animals were more heavily infested than the good or moderately conditioned small ruminants, while lactation was associated with an increase in faecal egg count. This observation is in agreement with findings of many other studies on nematode infections in small ruminants [20, 39, 40].

In this study, tethered animals presented with higher faecal egg counts than those reared on free range. Tethering of sheep and goats, a common practice in wet seasons in traditional husbandry systems, has been reported to result in increased contamination of pasture with infective larvae of nematodes such as *H. contortus* [29].

Where farmers were trained and therefore able to treat their animals by themselves, the worm burden was low in comparison to soliciting help from veterinarians. This observation reiterates the findings of Nampanzira et al. [41], where rural smallholder goat farmers ranked limited access to veterinary services among the four most important constraints to goat farming in Uganda, warranting the farmer's personal intervention by, among others, learning the basics of deworming.

### 4.3. Risk Factors Associated With the Presence of Haemonchosis in Small Ruminants in Apac District, Northern Uganda

Our results show that species of animal, breed, age, animal origin (subcounty), faecal consistency, lactation, grazing system, and anthelmintics use were important predictors of *H. contortus* infection in small ruminants. The association of *H. contortus* infection with animal breeds observed in this study has been documented previously [10, 42, 43]. These findings as well as our study show that indigenous small ruminants are more tolerant to worms than the exotic breeds or their crosses. The low levels of infection in indigenous breeds could be attributed to their ability to deter infection or tolerate certain levels of infection without showing susceptibility as compared to crossbreeds. Bishop [44] and Garedaghi and Bahavarnia [45] observed that genetic variation in resistance/resilience of breeds and immunity to helminth parasites are the most probable explanations. The high prevalence in exotic breeds or their crosses could also be due to their extensive pasture grazing pattern, which exposes them to high infestation levels compared to indigenous breeds. The result further showed that the infection rate was more prevalent in small ruminants with soft faeces than in those with normal faeces. This could be attributed to the fact that small ruminants with soft faeces had gastroenteritis or digestion/absorption disruption effects normally associated with haemonchosis. This could lead to a reduction in feed digestibility, chronic weight loss, and weakness. Furthermore, our results show that younger animals had a higher risk of haemonchosis than older animals. This is probably due to the fact that young animals tend to have low natural immunity compared to adults [46]. This is because immunological maturity occurs as animals grow old and there is an increase in acquired resistance due to repeated exposure to parasites [47].

In this study, small ruminants tethered had higher odds of infection than those freely grazed. It is known that tethering animals is associated with a high stocking rate, mixing of different species, and high pasture contamination than open grazing. Tethered animals are always confined; they eat the little available vegetation, usually on the ground, leading to an increased likelihood of picking infective *H. contortus* larvae from contaminated vegetation. This result agrees with the finding of Nsereko et al. [17] that small ruminants grazing freely in large open pastures, typically covered by woody vegetation and shrubs, have a limited likelihood of infection with *H. contortus* larvae. Furthermore, Kabasa, Opuda-Asibo, and ter Meulen [48] reported a lower worm burden in animals browsing on shrubs that could be due to a reduction in the intake of infective larvae from pasture close to the ground or the presence of browse plant species containing substantial amounts of crude protein and condensed tannins which are known to confer resistance to *H. contortus* [48].

The present study also showed that small ruminants not receiving antihelmintic treatment had a higher risk of haemonchosis than those receiving routine veterinary care (deworming). Badaso and Addis [19] demonstrated a positive correlation between haemonchosis infection and anthelmintic use. This finding is consistent with the current Ugandan livestock husbandry practices and agricultural policy, in which decisions about whether or not to prevent or treat infections are made at the household level (mostly based on economic considerations) and larger, coordinated preventive veterinary medical efforts are rare.

Finally, our results showed that multispecies grazing and haemonchosis increased the likelihood of *H. contortus* infection. The high number of other livestock on the farm could potentially act as reservoirs of infection for small ruminants. In addition, these other livestock are grazed together with small ruminants, leading to overgrazing and poor pasture which forces small ruminants to graze closer to the ground, resulting in the consumption of a higher number of infective stages of parasites [29].

In conclusion, the present study shows a relatively high infection rate in the study area that could be responsible for the loss of production and mortality. Species of animal, breed, age, animal origin (subcounty), faecal consistency, lactation, grazing system, and anthelmintics use were important predictors of *H*. *contortus* infection in small ruminants. Therefore, controlling *H. contortus* of small ruminants in the study area will require the use of an integrated approach that involves the proper use of anthelmintics and the institution of appropriate control measures that should necessarily include adopting better grazing management options such as rotational grazing to reduce larval contamination of vegetation and age-targeted deworming.

## Figures and Tables

**Table 1:** General characteristics of the households surveyed and the prevalence of haemonchosis in small ruminants.

**Variable**	**Category**	**Total number of animals examined**	**Copromicroscopy (faecal egg identification)**	**Coproculture (larvae in faecal sample)**	***χ*2**	**p** ** value**
			**Percentage positive (%)**	**Percentage positive (%)**		
Origin (subcounty), *n* (goats, sheep)	Nambieso	128 (65, 63)	78.1	68.8	12.858	**0.025**
	Chawente	134 (64, 70)	70.9	72.4		
	Akokoro	126 (66, 60)	74.6	70.6		
	Inomo	125 (62, 63)	73.6	89		
	Chegere	127 (62, 65)	80.3	70.4		
	Ibuje	128 (65, 63)	62.5	78.7		

Species	Goat	384	67.7	61.5	12.3	≤ **0.001**
	Sheep	384	78.9	79.7		

Breed	Local	683	72.4	70	3.997	**0.046**
	Cross	85	82.4	75.3		

Age	Adult	549	71	69.2	5.066	**0.024**
	Young	219	79	74		

Body condition	Good	254	72	70.5	5.341	0.069
	Medium	347	70.9	67.7		
	Poor	167	80.2	75.6		

Faecal consistency	Normal	399	66.2	62.7	21.647	≤ **0.001**
	Soft	369	81	79.1		

Grazing method	Freely grazing	259	66	59.8	10.596	**0.001**
	Tethering	509	77	76		

Multispecies grazing	No	440	66.6	61.1	23.75	≤ **0.001**
	Yes	328	82.3	83.2		

Anthelmintic use	Yes	264	63.3	60.2	20.764	≤ **0.001**
	No	504	78.6	76		

No. of animals kept	Less than 5	290	68.6	63.4	5.23	**0.022**
	More than 5	478	76.2	74.9		

Habouring other infections	Yes	207	70	67.1	1.538	0.215
	No	561	74.5	71.8		

Pregnancy	Yes	247	75.3	72.9	1.191	0.275
	No	323	71.2	69.3		

Lactation status	Yes	182	80.8	81.3	8.222	**0.004**
	No	388	69.3	66		

Sex of the animal	Male	198	74.2	69.7	0.119	0.73
	Female	570	73	70.9		

Agroecological zone	North Western Savannah	380	72.1	70.5	0.555	0.456
	Kyoga plains	388	74.5	70.6		

Size of grazing land	Less than 2	303	74.6	71.9	0.419	0.517
	More than 2	465	72.5	69.7		

Quality of pasture	Good	538	72.7	70.3	0.65	0.546
	Poor	230	74.8	71.3		

Farmers' level of education	No formal education	109	75.2	72.5	0.555	0.907
	Primary education	370	73.5	72.2		
	Secondary	230	71.7	67.8		
	Tertiary	59	74.6	67.8		

*Note:n* is the total number of small ruminants sampled per subcounty. Significant *p* values are presented in bold.

**Table 2:** Risk factors associated with *H. contortus* in small ruminants in Apac district, northern Uganda.

**Variable**	**Category**	**Prevalence**		**cOR, 95% CI**			**aOR, 95% CI**		
		**Total examined**	**Percentage positive (%)**	**Crude odds ratio**	**95% CI**	**p** ** value**	**Adjusted odds ratio**	**95% CI**	**p** ** value**
Origin (subcounty)	Nambieso	100	78.1	2.505	1.378–4.555	0.003	2.355	1.342–4.131	**0.003**
	Chawente	95	70.9	1.588	0.912–2.765	0.102	1.356	0.800–2.300	0.258
	Akokoro	94	74.6	1.889	1.053–3.389	0.033	1.867	1.079–3.231	**0.026**
	Inomo	92	73.6	1.641	0.916–2.941	0.096	1.657	0.960–2.859	**0.070**
	Chegere	102	80.3	3.208	1.733–5.939	≤ 0.001	2.504	1.409–4.452	**0.002**
	Ibuje	80	62.5	1					

Species	Goat	260	67.7	0.64	0.431–0.953	0.028	0.64	0.431–0.953	**0.028**
	Sheep	303	78.9	1					

Breed	Local	493	72.4	0.415	0.218–0.788	0.007	0.474	0.256–0.877	**0.017**
	Cross	70	82.4	1					

Age	Adult	390	71	1					
	Young	173	79	1.722	1.174–2.526	≤ 0.001	1.642	1.107–2.436	**0.014**

Faecal consistency	Normal	264	66.2	0.577	0.393–0.849	0.005	0.517	0.358–0.748	**≤ 0.001**
	Soft	299	81	1					

Lactation status	No	388	69.3	0.547	0.356–0.841	0.006	0.547	0.356–0.841	**0.006**
	Yes	182	80.8	1					

Grazing method	Freely grazing	171	66	1					
	Tethering	392	77	1.702	1.173–2.468	0.005	1.68	1.165–2.421	**0.005**

Grazing mixed species	No	293	66.6	0.427	0.302–0.603	≤ 0.001	0.407	0.286–0.580	**≤ 0.001**
	Yes	270	82.3	1					

Anthelmintic use	Yes	167	63.3	1					
	No	396	78.6	1.888	1.346–2.649	≤ 0.001	1.968	1.368–2.832	**≤ 0.001**

*Note:* Significant *p* values are presented in bold.

## Data Availability

The data that support the findings of this study are available from the corresponding author upon reasonable request.
